# Ablation of Lrp4 in Schwann Cells Promotes Peripheral Nerve Regeneration in Mice

**DOI:** 10.3390/biology10060452

**Published:** 2021-05-21

**Authors:** Tian-Kun Hui, Xin-Sheng Lai, Xia Dong, Hongyang Jing, Ziyang Liu, Erkang Fei, Wen-Bing Chen, Shunqi Wang, Dongyan Ren, Suqi Zou, Hai-Tao Wu, Bing-Xing Pan

**Affiliations:** 1 School of Life Sciences, Nanchang University, Nanchang 330031, China; sdhuitiankun@163.com (T.-K.H.); lxs19841@163.com (X.-S.L.); jinghongyangjhy@126.com (H.J.); nculzy2020@163.com (Z.L.); fek@ncu.edu.cn (E.F.); cwb100405@163.com (W.-B.C.); wsqi@ncu.edu.cn (S.W.); beautyin2011@126.com (D.R.); 2 Institute of Life Science, Nanchang University, Nanchang 330031, China; 18296111044@163.com; 3 School of Basic Medical Sciences, Nanchang University, Nanchang 330031, China; 4 Department of Neurobiology, Beijing Institute of Basic Medical Sciences, Beijing 100850, China; 5 Key Laboratory of Neuroregeneration, Co-Innovation Center of Neuroregeneration, Nantong University, Nantong 226019, China

**Keywords:** Schwann cells, Lrp4, nerve injury, regeneration, proliferation

## Abstract

**Simple Summary:**

Schwann cells (SCs) are the primary glial cells in the peripheral nervous system and play an indispensable role in peripheral nerve regeneration. A recent study indicated the potential involvement of low-density lipoprotein receptor-related protein 4 (Lrp4) in axonal regeneration in larval zebrafish. However, whether Lrp4 participates in nerve regeneration in mammals remains unknown. Herein, we constructed conditional knockout (cKO) mice in which Lrp4 was specifically deleted in SCs under the control of the Dhh-cre promoter. We found that the peripheral nerve regeneration in cKO mice was more robust than that in control mice. Assessment of SC proliferation via BrdU staining revealed significantly increased cell numbers in the cKO mice. Furthermore, as a master transcription factor participating in the onset of myelination, Krox-20 was dramatically downregulated in the injured nerves of the cKO group compared with the control group nerves. The enhanced demyelination speed in cKO mice was confirmed by electron microscopy. Altogether, these results suggest that Krox-20 downregulation in mutant mice leads to SC proliferation upon injury and that SCs can remove myelin debris, thereby promoting nerve regeneration.

**Abstract:**

Low-density lipoprotein receptor-related protein 4 (Lrp4) is a critical protein involved in the Agrin-Lrp4-MuSK signaling pathway that drives the clustering of acetylcholine receptors (AChRs) at the neuromuscular junction (NMJ). Many studies have shown that Lrp4 also functions in kidney development, bone formation, nervous system development, etc. However, whether Lrp4 participates in nerve regeneration in mammals remains unknown. Herein, we show that Lrp4 is expressed in SCs and that conditional knockout (cKO) of Lrp4 in SCs promotes peripheral nerve regeneration. In Lrp4 cKO mice, the demyelination of SCs was accelerated, and the proliferation of SCs was increased in the injured nerve. Furthermore, we identified that two myelination-related genes, Krox-20 and Mpz, were downregulated more dramatically in the cKO group than in the control group. Our results elucidate a novel role of Lrp4 in peripheral nerve regeneration and thereby provide a potential therapeutic target for peripheral nerve recovery.

## 1. Introduction

Peripheral nerve injuries are common (incidence of 1 in 1000 individuals per year) and result from systemic diseases or disabling diseases causing localized damager [1]. However, unlike injuries in the central nervous system (CNS) that result in loss of axon regeneration ability, the extra environment in the peripheral nervous system (PNS) permits axon regeneration in mammals [2]. Schwann cells (SCs) are considered to be indispensable for axon regeneration after nerve injury [3,4]. SCs in the PNS include myelinating SCs (mSCs) and nonmyelinating SCs (nmSCs). Following nerve injury, mSCs undergo demyelination and reprogramming into an immature form once they lose contact with axons; these cells are termed repair SCs (a subset of nmSCs). These repair cells proliferate quickly and activate a set of supportive repair programs, such as upregulating neurotrophic factors, activating myelin autophagy, and recruiting macrophages [3,4]. At the early stage of axon regeneration, these repair cell assemblies are enclosed by a basal lamina and form Büngner bands to provide a tissue bridge for axon clamping. Eventually, once axon regeneration is completed, the repair cells are spirally rewrapped into large-caliber regenerated axons to form either mSCs or dozens of small-caliber axons to create Remak SCs (a subset of nmSCs). Terminal SCs (tSCs) are another subset of nmSCs that are specifically located at neuromuscular junctions (NMJs). Many studies have shown that tSCs play critical roles in motor axon innervation, synaptogenesis, synaptic pruning, and cholinergic transmission in NMJ areas [5,6,7,8].

Low-density lipoprotein receptor-related protein 4 (Lrp4) is a member of the LDL receptor family and is most well-known to participate in the Agrin-Lrp4-MuSK signaling pathway, which drives AchR clustering on the postsynaptic membrane during NMJ development and maintenance [9,10,11]. To date, Lrp4 is known to be involved in a myriad of processes including bone formation [12,13], limb anomalies [14], craniofacial organogenesis [15], kidney malformations [16], and skin placode formation [17]. In the nervous system, Lrp4 participates in CNS development [18], cognitive function and plasticity [19], adult hippocampal neurogenesis [20], amyotrophic lateral sclerosis (ALS) [21], and Aβ clearance in Alzheimer’s disease [22]. Moreover, Lrp4 was shown to play a protective role in an ischemic brain injury mouse model [23]. Recently, Lrp4 was also found to critically serve as a retrograde signal to regulate presynaptic nerve terminal differentiation. The coculture of neurons and HEK293 cells overexpressing Lrp4 has shown that synaptic protein or SV2 aggregation is increased in axons [10]. Our published data also suggests that Lrp4 regulates presynaptic differentiation by directly binding to motor axons and inducing the aggregation of synaptic vesicles and active region proteins [9]. Although the overexpression of MuSK activity in Lrp4 knockout mice can restore AChR assembly and form NMJs, motor axons overgrow throughout the muscle and rarely stop at the AChR cluster [9]. These results suggest that Lrp4 may serve as a stop signal to prevent axon overgrowth [24]. However, a recent study reported that the knockout of Lrp4 in larval zebrafish could inhibit axon regeneration, while the specific deletion in the muscle and motor neurons could not rescue this defect [25]. The author concluded that SC-expressed Lrp4 may play a critical role in axon regeneration [25]. Considering that AChR prepatterning in zebrafish does not require Lrp4, motor axons display only slight overbranching in the absence of Lrp4 [26]. Thus, it is necessary to study whether Lrp4 plays a differentiation role in the progression of axon regeneration in mammalian and low-vertebrate animals.

In this study, we constructed conditional knockout (cKO) mice in which Lrp4 was specifically deleted in SCs under the control of the Dhh-cre promoter. We found that the peripheral nerve regeneration in cKO mice was more robust than that in control mice in nerve crush and nerve transplantation models. Based on the proliferation and demyelinating transcriptional expression of SCs, we conclude that deleting Lrp4 could promote axonal regeneration in the PNSs of mammalian species. Our results point to a novel role of Lrp4 in PNS regeneration and thereby provide a potential therapeutic target for peripheral nerve recovery after injury.

## 2. Materials and Methods

### 2.1. Generation and Genotyping of Mouse Lines

The *Lrp4-LacZ* mice were described previously [27,28]. Lrp4^flox/flox^ (*Lrp4^f/f^*) mice [9] were crossed with Dhh-cre mice (Jax stock #012929) to generate *Dhh::Cre*;*Lrp4^f/f^* mice (*Dhh-Lrp4^−/−^*, referred to as cKO hereafter). Rosa26-LSL-tdTomato mice were purchased from Jackson Laboratory (stock #021876); two-month-old male mice were used in the current study. The experimental procedures were approved by the Institutional Animal Care and Use Committee of the Nanchang University.

### 2.2. Reagents

Antibodies against the neurofilament (NF, C28E10) and synapsin (SYN, D12G5) were purchased from Cell Signaling Technology. The antibody against S100β (IR504) was purchased from Dako. CF568 α-bungarotoxin (#00006, 1:3000 for staining) was purchased from Biotium. CD68 (D4B9C) was purchased from Cell Signaling. AlexaFluor-488 goat anti-rabbit IgG (A-11034) and AlexaFluor-488 goat anti-mouse IgG (A-11001) were purchased from Invitrogen. β-galactosidase (β-Gal, SAB3500043) was purchased from Sigma. The remaining chemicals were purchased from Sigma-Aldrich unless otherwise stated. SYBR green mix (100029284) was purchased from Takara. A high-capacity cDNA Reverse Transcription kit (4368814) was purchased from ABI. In addition, μ-conotoxin GIIIB (H-9015.1000BA) was purchased from Bachem Americas.

### 2.3. Immunofluorescence

Muscles were fixed in 4% paraformaldehyde (PFA) at room temperature for 30 min and incubated with 0.1 M glycine for 30 min. Next, the tissue sections were incubated with blocking buffer (2% BSA, 7% goat serum, and 0.5% Triton X-100 in PBS) for 2–4 h at room temperature or overnight at 4 °C. After washing three times each for 15 min, the samples were incubated with the primary antibodies in blocking buffer overnight at 4 °C. The tissues were washed three times and incubated with fluorescently labeled secondary antibodies for 2–4 h at 25 °C. After washing three times for 30 min in PBS, the tissues were flat-mounted using Hydromount (National Diagnostics, Kolkata, India). For sciatic nerve staining, the sciatic nerves were embedded in OCT. The frozen samples were cut into 20-µm-thick sections, fixed in 4% PFA, and washed in PBS at room temperature for 30 min. Next, the sections were incubated in blocking buffer (2% BSA, 7% goat serum, and 0.5% Triton X-100 in PBS) for 2–4 h at room temperature, and then incubated overnight with primary antibodies at 4 °C. After washing three times with PBS, the samples were incubated at room temperature for 2–4 h with the Alexa-488 goat anti-rabbit or Alexa-594 goat anti-mouse secondary antibody. The samples were then washed three times and mounted in Hydromount (National Diagnostics, Kolkata, India). For all NMJ staining, α-BTX was added to the secondary antibody solution. For *Dhh-tdTomato* and *Dhh-tdTomato-Lrp4^−/−^* mouse NMJ staining, CF568-conjugated α-BTX was replaced with CF488-conjugated α-BTX. Images were collected using florescence microscope (FSX100, Olympus, Tokyo, Japan), and image analysis and NMJ counting were conducted using ImageJ.

### 2.4. Western Blot Analysis

Western blotting was performed as previously described [11]. Briefly, the sciatic nerves were homogenized and lysed in RIPA buffer. After centrifugation the supernatant was collected, and the lysate protein concentrations were measured. Protein samples (50 μg of protein) were separated by SDS-PAGE and transferred onto nitrocellulose membranes. The membrane was incubated in 5% milk and then incubated with the primary antibodies, anti-LRP4 (1:1000, clone N207/27, UC Davis/NIH NeuroMab Facility) and anti-GAPDH (1:2000, HRP-60004, Abcam, Cambridge, United Kingdom). After washing, the membranes were incubated with the anti-rabbit or anti-mouse horseradish peroxidase (HRP)-conjugated secondary antibody (1:2000, Invitrogen, Carlsbad, CA, USA), and the signal was detected by Western HRP chemiluminescence reagents (Thermo, Waltham, MA, USA). The grayscale values of the bands were quantified using ImageJ.

### 2.5. BrdU Staining

Bromodeoxyuridine (BrdU) staining was performed as previously described with modifications [29]. The mice were injected with BrdU (B5002 Sigma-Aldrich, St. Louis, MO, USA) at 5 days post crush (dpc, 100 mg/kg, three times at 6 h intervals). Two hours after the final injection, the mouse hearts were perfused with 4% PFA. Sciatic nerves were embedded in OCT compound, and longitudinal sections were cut into 20-µm-thick sections. The samples were rinsed with 0.5% Triton X-100 for 30 min and incubated with 2 N HCl for 1 h at 37 °C. The samples were then rinsed with borate buffer (0.1 M, pH 8.5) for 8–12 min and washed with PBS three times. Next, the samples were blocked in blocking buffer (2% BSA, 7% goat serum, and 0.5% Triton X-100 in PBS) for 2–4 h at room temperature. The samples were then immunostained with an anti-BrdU antibody (1:1000 in blocking buffer) overnight at 4 °C. After washing 3 times for 15 min each with 0.5% Triton X-100 in PBS, the tissues were incubated with an Alexa Fluor 488-conjugated antibody and DAPI overnight at 4 °C. After washing 3 times for 30 min each with 0.5% Triton X-100 in PBS, the tissues were flat-mounted in Hydromount (National Diagnostics, Atlanta, GA, USA).

### 2.6. TUNEL Staining

Terminal deoxynucleotidyl transferase dUTP nick end (TUNEL) staining was performed using a TUNEL apoptosis detection kit (Sigma 11684795910) according to the manufacturer’s protocol with some modifications. At 5 dpc, the tissues were sectioned and fixed with 4% PFA for 1 h at room temperature, after which the sciatic nerves were embedded in OCT compound and longitudinal sections were cut into 20-µm-thick sections. The samples were washed three times with PBS for 15 min each time and then permeabilized with 0.5% Triton X-100 in PBS for 2 h at room temperature. The samples were then incubated with a TUNEL reaction mixture for 60 min at 37 °C and rinsed with DAPI for 30 min. After washing with PBS three times for 15 min each time, the tissues were mounted in Hydromount (National Diagnostics).

### 2.7. NMJ Electrophysiology

NMJ electrophysiological analysis was performed as previously described [9,30]. Mouse diaphragms with intact phrenic nerves were dissected in oxygenated (95% O_2_, 5% CO_2_) Ringer’s solution (136.8 mM NaCl, 5 mM KCl, 12 mM NaHCO_3_, 1 mM NaH_2_PO_4_, 1 mM MgCl_2_, 2 mM CaCl_2_, and 11 mM D-glucose, pH 7.3) and mounted on Sylgard gel in a dish perfused with the oxygenated Ringer’s solution. The muscle fiber center was pierced with microelectrodes (20–50 MΩ, filled with 3 M KCl, resting potential ranging from −65 and −80 mV) to record miniature endplate potentials (mEPPs). Ten recordings were performed, with each lasting approximately 3 min. For the EPPs, the phrenic nerves were stimulated by a suction electrode. Muscle contraction was blocked by the addition of 2.5 μM μ-conotoxin when phrenic nerves were stimulated. Data were collected with a MultiClamp 700B amplifier (Molecular Devices, Sunnyvale, CA, USA), digitized (10 kHz low-pass filtered) with Digidata 1550A (Molecular Devices, Sunnyvale, CA, USA), and analyzed using Clampfit 10.5 software (Molecular Devices, Sunnyvale, CA, USA).

### 2.8. Denervation Experiment

For the nerve crush experiments, mice (male, 2 months old) were anesthetized, and their right thigh fur was shaved and sterilized with 75% alcohol. A 1 cm incision was made in the skin over the lateral femur. The sciatic nerves at the mid-thigh were exposed and clamped with micro forceps for 10 s. The incisions were closed with steel clips, and the mice were placed on a thermostatic rubber pad and kept warm until their revival. In the nerve transplant experiment, sciatic nerve segments (approximately 1 cm long) were removed from the control and cKO mice and exchanged between the mice. The nerve segments were sutured to the existing nerve at both ends in the correct orientation. After 8 weeks, the soleus was isolated and stained for NMJs.

### 2.9. Biotin Tracing

At 7 dpc, the mice were anesthetized, the sciatic nerve was removed from the injury site, and the distal stump was incubated with biotin. Two hours after incubation, the mice were perfused with 4% PFA. The sciatic nerve cryosections were labeled with streptavidin Alexa 568 for 1 h at room temperature. After washing with PBS 3 times, the sections were mounted with Hydromount (National Diagnostics).

### 2.10. Quantitative Real-Time PCR (qRT-PCR)

Total RNA was isolated from the sciatic nerve using TRIzol reagent (15596-026, Invitrogen, Carlsbad, CA, USA), and 2 μg of total RNA was reverse transcribed into cDNA with High capacity cDNA Reverse Transcription kit. The product was used as a template for qPCR, which was performed using a SYBR Green detection kit (K0222, Fermentas, Glen-Burnie, MD, USA), and the total reaction volume was 20 μL. The primers were as follows: Lrp4, 5′-GTG TGG CAG AAC CTT GAC AGT C-3′ (forward) and 5′-TAC GGT CTG AGC CAT CCA TTC C-3′ (reverse); c-Jun, 5′-CAG TCC AGC AAT GGG CAC ATC A-3′ (forward) and 5′-GGA AGC GTG TTC TGG CTA TGC A-3′ (reverse); A-Raf, 5′-GCG GAA GTC CTT GGC AGA TGA A-3′ (forward) and 5′-CCG AAA CAC AGT GCC AAA AGA GC-3′ (reverse); B-Raf, 5′-CGC CAA GTC AAT CAT CCA CAG AG-3′ (forward) and 5′-CAC CGA GAT TTC ACT GTG GCT AG-3′ (reverse); C-Raf, 5′-CTT CAG GAA CGA GGT GGC TGT T-3′ (forward) and 5′-TGC TGC CTT CAC ACC ACT GAG T-3′ (reverse); Mpz, 5′-CTG CTC CTT CTG GTC CAG TGA A-3′ (forward) and 5′-AGG TTG TCC CTT GGC ATA GTG G-3′ (reverse); Krox-20, 5′-CCT TTG ACC AGA TGA ACG GAG TG- 3′ (forward) and 5′-CTG GTT TCT AGG TGC AGA GAT GG-3′ (reverse); NF-κB, 5′-GCA GCA CTA CTT CTT GAC CAC C-3′ (forward) and 5′-TCT GCT CCT GAG CAT TGA CGT C-3′ (reverse); Notch, 5′-GCT GCC TCT TTG ATG GCT TCG A-3′ (forward) and 5′-CAC ATT CGG CAC TGT TAC AGC C-3′ (reverse); Sox-2, 5′-AAC GGC AGC TAC AGC ATG ATG C-3′ (forward) and 5′-CGA GCT GGT CAT GGA GTT GTA C-3′ (reverse); Id-2, 5′-TCA CCA GAG ACC TGG ACA GAA C-3′ (forward) and 5′-TGC TAT CAT TCG ACA TAA GCT CAG-3′ (reverse); GAPDH, 5′-CAT CAC TGC CAC CCA GAA GAC TG-3′ (forward) and 5′-ATG CCA GTG AGC TTC CCG TTC AG-3′ (reverse). The amplification programming standards were performed as previously described, and each sample was measured in triplicate. The mRNA levels were normalized to those of GAPDH.

### 2.11. Electron Microscopy

At 7 dpc, the mice were anesthetized and cardiac perfused with 4% PFA and 2% glutaraldehyde in 0.1 M sodium cacodylate buffer (NaCac, pH 7.4). The distal segments of the sciatic nerves were separated and fixed overnight at 4 °C in the same perfusion fixative for 24 h. Electron microscopy (EM) analysis of myelin was performed as described previously with modifications [29]. Ultrathin sections were photographed with an electron microscope.

### 2.12. Statistical Analysis

Before being analyzed, all data in our study were checked by the D’Agostino–Pearson omnibus. Data were analyzed by the unpaired *t*-test, one-way ANOVA, and two-way ANOVA using GraphPad Prism. Data are shown in dot plots as the mean ± SEM. The significance criterion was set at *p* < 0.05. *, **, ***, and **** represent *p* < 0.05, *p* < 0.01, *p* < 0.001, and *p* < 0.0001, respectively.

## 3. Results

### 3.1. Lrp4 Expression in SCs

**Figure 1:**
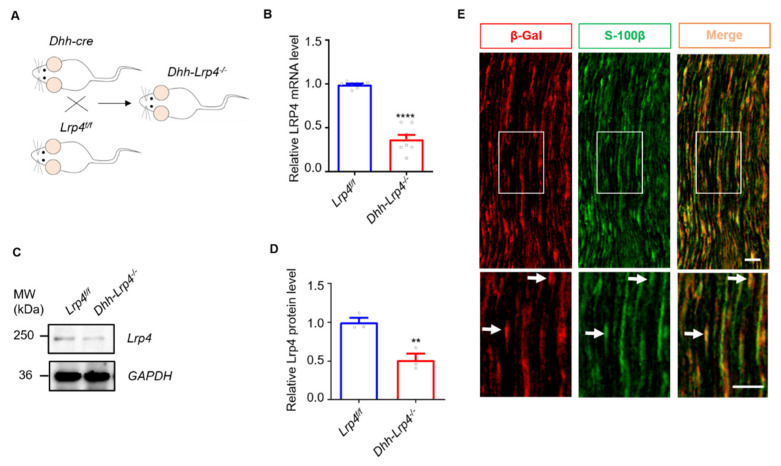
Lrp4 in SCs was successfully deleted in *Lrp4 cKO* mice. (**A**) Diagram of the strategy used to generate the *Lrp4 cKO* mice. (**B**) Reduced Lrp4 mRNA levels in cKO mice compared with control mice. **** *p <* 0.0001, control and cKO, 8 mice/group. (**C**,**D**) Reduced Lrp4 protein levels in cKO mice compared with control mice. ** *p* = 0.0083, 3 mice/group. (**E**) The images show that Lrp4 (red) is localized in SCs (green). The white arrows indicate the costaining of β-Gal and S-100β. *Scale bar* = 50 μm.

Previous studies have demonstrated the function of Lrp4 in the glial cells of the CNS [22,23,27]. To initially investigate the function of Lrp4 in SCs, we generated SC *Lrp4 cKO* mice (*Dhh-Lrp4^−/−^*) by crossing *Lrp4^f/f^* mice with *Dhh::Cre* mice (Figure 1A). The sciatic nerves of the control (*Lrp4^f/f^*) and *Lrp4 cKO* mice were isolated for qRT-PCR and Western blotting. As shown in Figure 1B–D, both the mRNA and protein levels of Lrp4 were dramatically decreased in the *Lrp4 cKO* mice compared with the control mice. These results suggest that Lrp4 was successfully deleted from the SCs of *Lrp4 cKO* mice. Next, we examined the expression of Lrp4 in SCs from *Lrp4-LacZ* mice [27]. Exon 2 through 31 within the Lrp4 gene were replaced with LacZ, which encodesβ-gal. We found that β-gal was costained with the SC marker S100β, suggesting that Lrp4 was expressed in SCs (Figure 1E).

### 3.2. Nerve Regeneration Promotion in the Lrp4 cKO Mice

**Figure 2:**
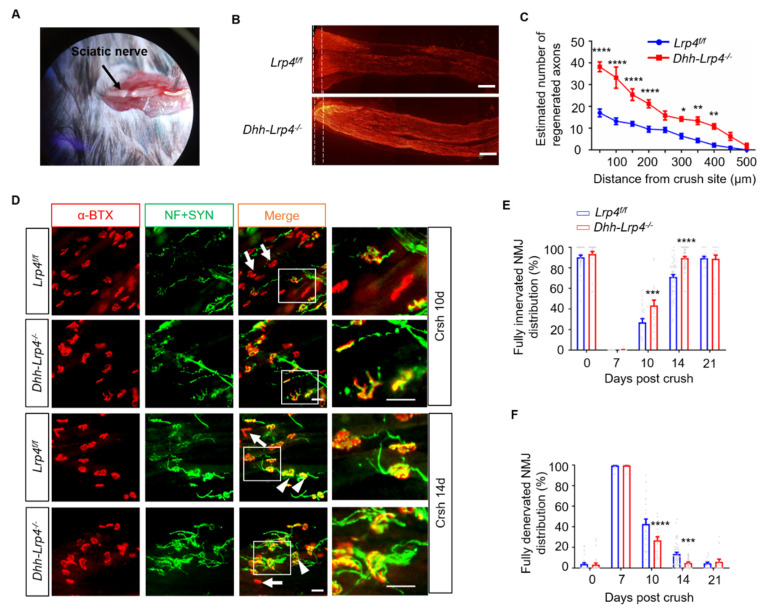
Ablation of Lrp4 in SCs promotes nerve regeneration. (**A**) Representative images of the sciatic nerve after crush surgery. (**B**) Biotin staining of sciatic nerve sections from cKO mice revealed more regenerated axons at 7 dpc. *Scale bar* = 200 μm. (**C**) The regenerated axons were longer in the cKO mice than in the control mice. **** *p* < 0.0001, ** *p* < 0.01, * *p* < 0.05, control, n = 6 slices; cKO, n = 5 slices, 3 mice/group. (**D**) Representative images of NMJ regeneration at 10 and 14 dpc. The arrowheads indicate fully reinnervated NMJs. The arrows indicate denervated NMJs. *Scale bar* = 50 μm. (**E**) Quantification of the fully reinnervated endplates at 0, 7, 10, 14, and 21 dpc. Control mice (26.9 ± 3.6% and 71.0 ± 2.4% at 10 and 14 dpc, respectively) display fewer reinnervated NMJs at 10 and 14 dpc than the cKO mice (43.4 ± 5.0% and 89.4 ± 1.5% at 10 and 14 dpc, respectively). *** *p* < 0.001, **** *p* < 0.0001, control, n = 33, 26, 23, 50, and 18 slices at 0, 7, 10, 14, and 21 days, respectively. cKO, n = 27, 22, 18, 50, and 16 slices at 0, 7, 10, 14, and 21 days, respectively. Three or four mice per group. (**F**) The fully denervated NMJs were reduced in cKO mice (26.7 ± 3.5% and 5.0 ± 0.9% at 10 and 14 dpc, respectively) 10 and 14 dpc compared with the control mice (42.6 ± 4.9% and 13.5 ± 1.5% for 10 and 14 dpc, respectively). **** *p* < 0.0001, *** *p* < 0.001, 3 or 4 mice per group. All the data are presented as mean ± SEM.

We previously showed that the motor axons can bypass AChR clusters to “overshoot” towards the periphery of muscle fibers during development in muscle-specific Lrp4 KO mice, implying that Lrp4 deficiency may promote axon growth [9]. Herein, to evaluate whether the ablation of Lrp4 in SCs promotes axon regeneration, we performed anterograde labeling with biocytin to trace the nerve axon regrowth trajectory at 7 dpc. Nerve crush was performed, as shown in Figure 2A. Significantly, the biotin-labeled NF were longer in Lrp4 cKO mice than in the control group (Figure 2B, the dotted line indicates the crush site). Moreover, the regenerated nerve numbers at different distances from the crush site were increased (Figure 2C), suggesting that Lrp4 expressed on mSCs may play a negative role in PNS regeneration. NMJ reinnervation is an excellent model for investigating whether regenerated axons completely retarget muscle fibers. At different days after nerve crush, the muscles were isolated and stained with CF568-conjugated α-BTX to visualize the AChR clusters and stained with antibodies against NF and SYN to visualize the axons and nerve terminals. We compared the ratios of regenerated NMJs in control and cKO mice. Depending on the overlapping extents of the nerve terminal and AChR cluster, the endplates were categorized as either completely reinnervated (the AChR clusters fully overlapped with the regenerated nerve terminals) or fully denervated (AChR clusters did not overlap with the regenerated nerve terminals). Accordingly, the completely reinnervated NMJ ratios in cKO mice were noticeably increased at 10 and 14 dpc compared with those of control mice (Figure 2D,E). The completely denervated NMJs in the *Lrp4 cKO* mice were significantly decreased at 10 and 14 dpc (Figure 2F). These results suggest that the deletion of Lrp4 in SCs promotes axon regeneration in the PNS.

### 3.3. The mSCs Rather Than tSCs Are Involved in Accelerating Nerve Regeneration in Lrp4 cKO Mice

**Figure 3:**
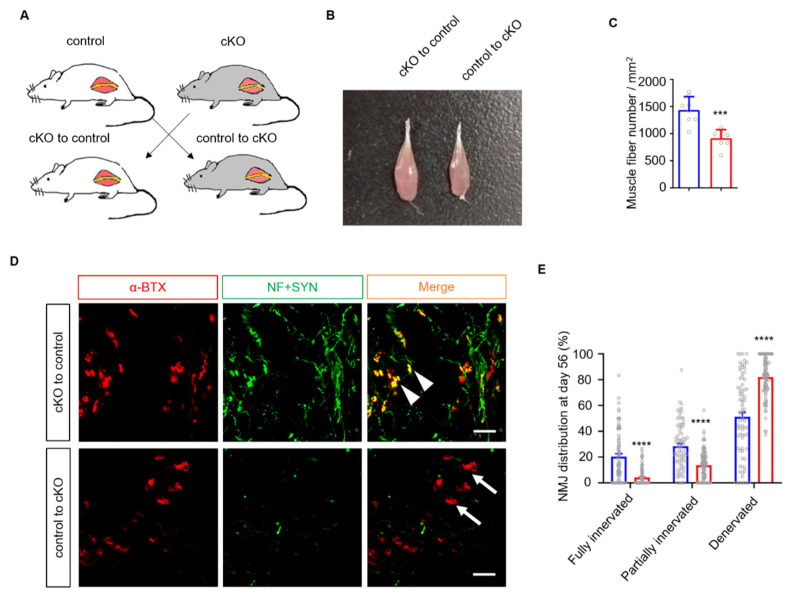
mSCs are involved in accelerating regeneration in *Lrp4 cKO* mice. (**A**) Schematic representation of the nerve exchange experiment. (**B**) The TA muscles in the cKO mice exhibited amyotrophy at 56 days after the transplant surgery. (**C**) Decreased TA muscle fiber numbers in control mice (1437.0 ± 86.9) compared with cKO mice (916.4 ± 55.9). Control and cKO, n = 8 slices, 3 mice/group, *** *p* = 0.0002. (**D**) Representative images of NMJ regeneration at 56 days after nerve transplant. The arrowheads indicate fully reinnervated NMJs. The arrows indicate denervated NMJs. *Scale bar* = 100 μm. (**E**) Reduced fully reinnervated and increased denervated endplates in cKO after nerve transplantation. **** *p* < 0.0001, control, n = 75 slices; cKO, n = 93 slices, 6 mice/group. The fully innervated NMJ percentage was 20.3 ± 2.3% in the control mice, compared with 4.2 ± 0.7% in the cKO mice; the partially innervated NMJ percentage was 28.5 ± 2.1% in the control mice, compared with 13.7 ± 1.2% in the cKO mice; and the denervated NMJ percentage was 51.2 ± 3.6% in the control mice, compared with 82.0 ± 1.6% in the cKO mice. All the data are presented as the mean ± SEM.

Both mSCs and tSCs are involved in peripheral nerve regeneration, as mSCs promote axonal regeneration, while tSCs guide the nerve terminal to NMJs. To verify the role of mSCs, we performed a nerve transfer experiment, which is the gold standard for assessing mSC function [29] (Figure 3A). We isolated the sciatic nerve from the control and cKO mice and exchanged the nerve segments. In this experiment, nerve allografts provided SCs from donor mice to host mice. Eight weeks after nerve transfer, the tibialis anterior (TA) muscles in the control mice appeared to display less relative atrophy than those from cKO mice (Figure 3B), which was confirmed by quantifying the number of muscle fibers (Figure 3C). We next quantified the number of reinnervated endplates in soleus muscles. As expected, the control mice displayed more reinnervated NMJs than the cKO mice (Figure 3D,E). These results demonstrate that the ablation of Lrp4 in mSCs provides a favorable environment for axon regrowth.

**Figure 4:**
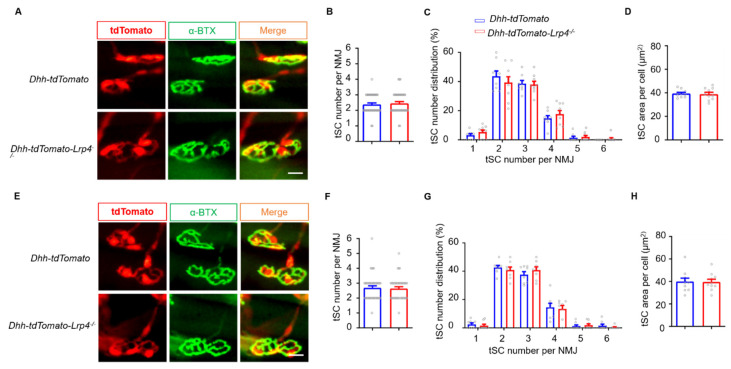
tSCs are not involved in accelerating regeneration in *Lrp4 cKO* mice. (**A**) Representative images of tSCs in *Dhh-tdTomato* and *Dhh-tdTomato-Lrp4^−/−^* mice at 7 dpc. *Scale bar* = 25 μm. (**B**) The number of tSCs per NMJ in the soleus muscle at 7 dpc. Control and cKO, n = 42 NMJs, 3 mice/group. (**C**) Distribution of the different tSC numbers in the NMJs of the soleus muscle at 7 dpc. Control and cKO, n = 8 slices. (**D**) The average area of the tSC in the soleus muscle at 7 dpc. Control and cKO, n = 10 cells. (**E**) Representative images of tSCs in *Dhh-tdTomato* and *Dhh-tdTomato-Lrp4^−/−^* mice under normal conditions. *Scale bar* = 25 μm. (**F**) The number of tSCs per NMJ in the soleus muscle. Control and cKO, n = 42 NMJs, 3 mice/group. (**G**) Distribution of the different tSC numbers in the NMJs of the soleus muscle. Control, n = 8 slices; cKO, n = 9 slices. (**H**) The average area of the tSC per NMJ in the soleus muscle. Control and cKO, n = 10 cells. All the data are presented as the mean ± SEM.

To determine whether Lrp4 ablation in tSCs is involved in promoting NMJ regeneration, we characterized the morphology of tSCs by generating *Dhh-tdTomato* and *Dhh-tdTomato-Lrp4^−/−^* mice whose SCs were labeled by red fluorescence. At 7 dpc, muscles were isolated and stained for NMJs. We found that each NMJ was covered by 2~4 tSCs, consistent with the tSC number under normal conditions (Figure 4A) [31,32]. In addition, the average tSC numbers, NMJ tSCs distribution, and tSC soma areas did not differ between the *Dhh-tdTomato* and *Dhh-tdTomato-Lrp4^−/−^* mice (Figure 4B–D). Furthermore, we investigated whether Lrp4 ablation influences tSCs under normal conditions (Figure 4E). Consistently, the average tSC number and distribution of tSCs in each NMJ and soma area were comparable between the *Dhh-tdTomato* and *Dhh-tdTomato-Lrp4^−/−^* mice (Figure 4F–H). In summary, these results suggest that the deletion of Lrp4 in mSCs but not in tSCs contributes to accelerating NMJ regeneration.

### 3.4. Enhanced SC Proliferation in Lrp4 cKO Mice after Nerve Damage

**Figure 5:**
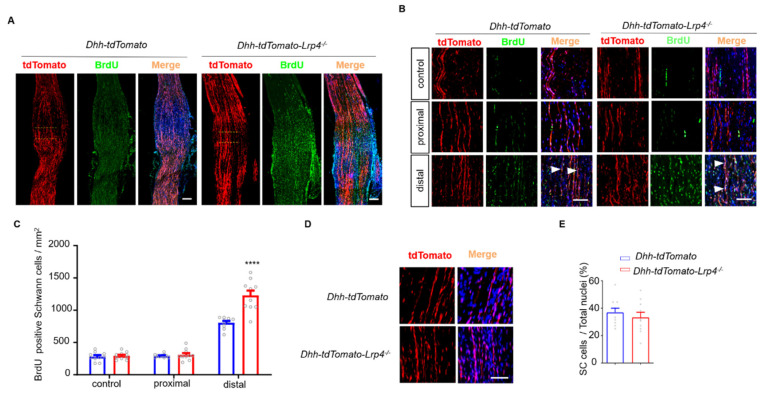
The proliferation of SCs in *Lrp4 cKO* mice was enhanced after nerve damage. (**A**) Immunostaining of the sciatic nerve of newly formed SCs at 5 dpc. *Scale bar* = 200 μm. (**B**) Representative images of the sham operation control and proximal and distal crushed nerves. The white arrows indicate the costaining of *Dhh-tdTomato* and BrdU. *Scale bar* = 50 μm. (**C**) Enhanced proliferation of SCs in *Dhh-tdTomato-Lrp4^−/−^* mice (1229.0 ± 75.2) sciatic nerves compared with *Dhh-tdTomato* mice (806.6 ± 27.6) after nerve crush. **** *p* < 0.0001, control and cKO, n = 10 slices, 3 mice/group. (**D**) Representative images of mSCs under normal conditions. *Scale bar* = 50 μm. (**E**) The mSC numbers under normal conditions did not differ between *Dhh-tdTomato* and *Dhh-tdTomato-Lrp4^−/−^* mice. Control, n = 20 slices; cKO, n = 21 slices, 3 mice/group. All the data are presented as the mean ± SEM.

**Figure 6:**
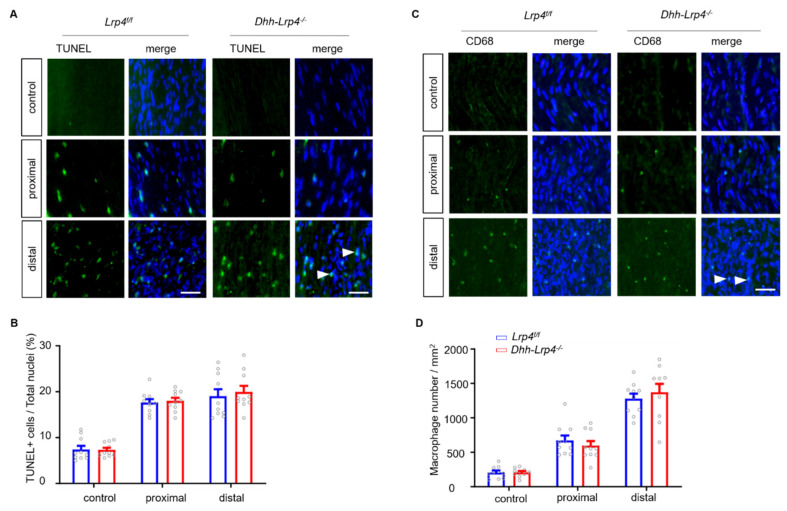
Lrp4 ablation in SCs does not influence SC survival or macrophage recruitment after injury. (**A**) The numbers of TUNEL-positive cells in the sham control and proximal and distal ends of crushed nerve segments did not differ between the control and cKO mice. The white arrows indicate the apoptotic cells. *Scale bar* = 25 μm (**B**) The proportion of TUNEL-positive cells was not significantly different between the control and cKO mice. Control and cKO, n = 10 slices, 3 mice/group. (**C**) Representative pictures of macrophages from control and cKO mice. The macrophage numbers in the sham control and proximal and distal ends of the crushed nerve segments did not differ between the control and cKO mice. The white arrows indicate the macrophages. *Scale bar* = 25 μm (**D**) Quantification of the macrophage numbers revealed no significant difference between the control and cKO mice. Control and cKO, n = 10 slices, 3 mice/group.

To explore the potential mechanisms, we detected SC proliferation and survival using the BrdU labeling and TUNEL assays, respectively. In *Dhh-tdTomato* or *Dhh-tdTomato-Lrp4^−/−^* mice, the right sciatic nerve was crushed while the left sciatic nerve was shammed to serve as the control. The mice were injected with BrdU 3 times at 5 dpc. Two hours after the final injection, the injured nerve segments and sham controls, including those at the proximal, distal, and injury sites, were isolated intact and then stained with the anti-BrdU antibody. As shown in Figure 5A, the number of BrdU+/Dhh+ cells, indicating newly formed SCs, was increased in the cKO mice. The dotted line indicates the crush site. Moreover, the SCs in cKO mice mainly proliferated in the distal nerve segments (Figure 5B,C), which is consistent with previous reports [33]. Furthermore, the numbers of SCs under normal conditions were comparable between the cKO and control mice, suggesting that the accelerated proliferation of SCs in the cKO mice was nerve injury dependent (Figure 5D,E). To determine whether the ablation of Lrp4 affects SC survival after injury, we used the TUNEL assay to detect apoptotic cells. As shown in Figure 6A,B, the numbers of TUNEL-positive cells did not differ between the control and cKO mice. These results suggest that Lrp4 deficiency in SCs leads to enhanced SC proliferation without disturbing SC survival after nerve injury.

During injury-induced SC reprogramming, macrophages are gradually recruited by SCs and cooperate with SCs to degrade myelin debris and thereby facilitate axonal regrowth [34,35]. To determine whether the increased SCs recruited more macrophages in cKO mice, we labeled the macrophages with an anti-CD68 antibody (Figure 6C), and the CD68+ cell numbers were comparable between control and cKO mice (Figure 6D). Taken together, these results suggest that the advantageous proliferation of SCs plays a potential role in promoting NMJ regeneration in *Lrp4 cKO* mice.

### 3.5. Lrp4 Deficiency Promotes Demyelination and Downregulates Krox-20 after Nerve Injury

**Figure 7:**
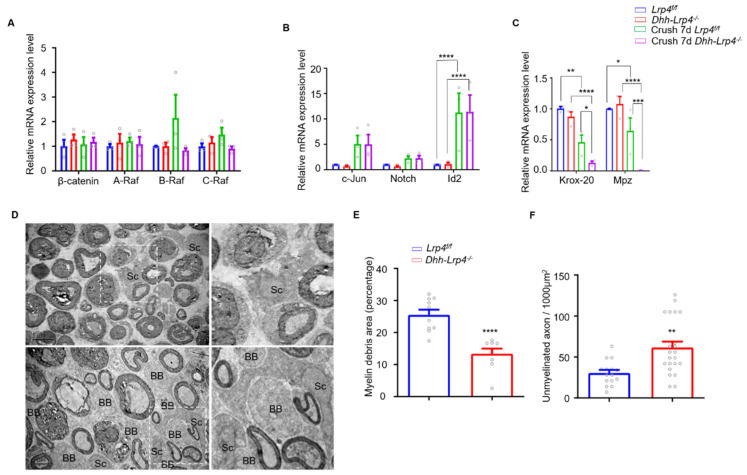
Lrp4 deficiency promotes demyelination and downregulates Krox-20 after nerve injury. (**A**) The β-catenin and MAPK pathway-related mRNA levels did not differ between the control and cKO mice (3 mice/group). (**B**) The mRNA levels of c-Jun, Notch, and Id2 were comparable between the control and cKO mice before injury and at 7 dpc. **** *p* < 0.0001 (3 mice/group). (**C**) The mRNA levels of Mpz and Krox-20 were decreased in the cKO mouse sciatic nerves compared with the control mice at 7 dpc. * *p* < 0.05, ** *p* < 0.01, *** *p* < 0.001, **** *p* < 0.0001 (3 mice/group). (**D**) Representative electron micrograph of control and cKO sciatic nerves. The cKO mice showed lower amounts of myelin debris and more regenerated axons. BB indicates the Büngner band, Sc indicates Schwann cells. *Scale bar* = 5 μm. (**E**) The size of the myelin debris area was decreased in the cKO mice (13.4 ± 1.6) compared with the control mice (25.5 ± 1.1). **** *p* < 0.0001, control, n = 10 slices; cKO, n = 9 slices (3 mice/group). (**F**) The number of newly formed axons was increased in cKO mice (61.5 ± 7.3) compared with control mice. (30.4 ± 3.9). ** *p* = 0.0021, control, n = 15 slices; cKO, n = 22 slices (3 mice/group). All the data are presented as the mean ± SEM.

To investigate the mechanisms underlying LRP4 deficiency-induced SC proliferation, we focused on the Wnt and MAPK signaling pathways, which are involved in promoting the proliferation of SCs. As shown in Figure 7A, before and after nerve injury, the mRNA levels of β-catenin and MAPK were comparable between the control and *Lrp4 cKO* mice. Next, we focused on the factors regulating SC demyelination processes. After nerve injury, SCs undergo demyelination rapidly, and demyelinated SCs proliferate to promote nerve regeneration. Substantial progress has been identifying the intrinsic factors in SCs that participate in this process [36,37]. c-Jun, Notch, and Id2 are positive regulators of SC demyelination [38]. However, our qRT-PCR results showed no differences in their levels in control and cKO mice under both normal and nerve damage conditions (Figure 7B). Next, we detected a negative factor related to demyelination, Krox-20, which is essential for SC demyelination after nerve injury [36,39]. Surprisingly, Krox-20 expression was significantly decreased in the mutant SCs compared with the control SCs. Accordingly, the expression of Mpz, which is required for proper myelin formation and maintenance, was reduced in the injury-induced cKO SCs compared with the control (Figure 7C). This result suggests that the decreased Krox-20 and Mpz levels in the mutant SCs may have enhanced SC demyelination after nerve injury. Furthermore, the nerve segments were subjected to EM analysis. Although myelin breakdown or collapse was apparent in both cKO mice and control mice seven days after injury, the cKO mice displayed more demyelination and a lower amount of myelin debris (Figure 7D). Statistically, the size of the myelin debris area was dramatically decreased (Figure 7E), and the regenerated axon numbers were increased in the cKO mice (Figure 7F). These results suggest rapid demyelination and myelinophagy upon Lrp4 deletion in SCs.

### 3.6. Normal NMJ Formation and Transmission in SC Lrp4 cKO Mice

**Figure 8:**
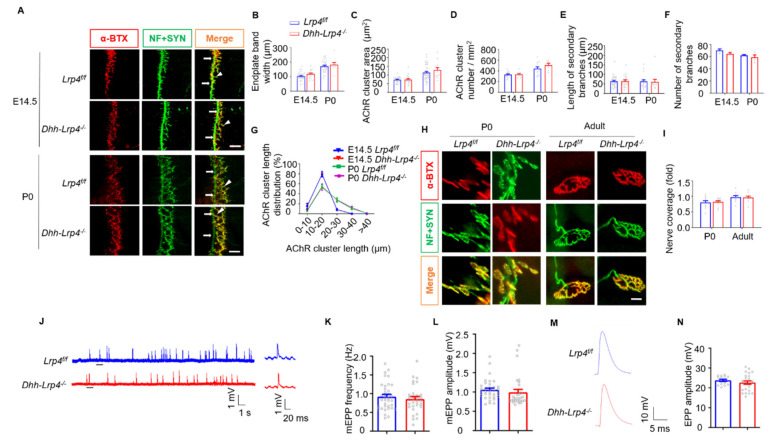
Lrp4 deficiency in SCs does not influence NMJ formation or transmission. (**A**) Representative images of the diaphragms of control and cKO mice at E14.5 and P0. The left ventral regions of the diaphragms of control and mutant mice at P0 and E14.5 were stained in whole-mount NMJ preparations. The arrow indicates the primary branch. The arrowhead indicates the secondary branch. *Scale bar* = 50 μm. (**B**) Comparable endplate bandwidths between the control and cKO mice. Control, n = 29 or 31 regions; cKO, n = 9 or 16 regions, 3 to 5 mice/group. (**C**) Comparable AChR cluster areas between the control and cKO mice. Control, n = 26 or 39 regions; cKO, n = 19 regions, 3 to 5 mice/group. (**D**) Comparable AChR cluster numbers between the control and cKO mice. Control, n = 12 or 15 regions; cKO, n = 11 regions, 3 to 5 mice/group. (**E**) Comparable secondary branch lengths between the control and cKO mice. Control, n = 29 or 22 branches; cKO, n = 21 branches, 3 to 5 mice/group. (**F**) Comparable secondary branch numbers between the control and cKO mice. 3 or 4 mice/group. (**G**) Comparable AChR cluster length distribution between the control and cKO mice of the same age. Control, n = 5 slices; cKO, n = 4 slices, 3 to 5 mice/group. (**H**) Enlarged images of a single NMJ from the limb muscle. *Scale bar* = 10 μm. (**I**) Comparable nerve coverage ratios between the control and cKO mice. Control and cKO, n = 10 NMJs, 3 or 4 mice/group. (**J**) Representative mEPP traces of adult control and cKO mice. (**K**,**L**) The mEPP frequencies and amplitudes were similar between the control and cKO mice. Control, n = 36 regions of different NMJs; cKOs, n = 30 regions of different NMJs, 3 or 4 mice/group. (**M**) Representative EPP traces in control and cKO mice. (**N**) The EPP amplitudes were similar in control and cKO mice. Control, n = 16 regions of different NMJs; cKOs, n = 25 regions of different NMJs, 3 or 4 mice/group. All the data are presented as the mean ± SEM.

Having demonstrated that the deletion of Lrp4 in mSCs promotes NMJ regeneration, we next examined whether SC-derived Lrp4 is required for NMJ development. We examined NMJs in the diaphragm muscles at E14.5 (initial NMJ formation) and P0 (during NMJ development) as previously reported [30]. The diaphragm and TA muscles were stained in whole-mount preparations with antibodies against NF and SYN to label axons and nerve terminal synaptic vesicles and with CF568-conjugated α-BTX to visualize the postsynaptic AChR clusters. The NMJ morphology in the cKO mice was similar to that of the control littermates at E14.5 and P0 (Figure 8A). The endplate bandwidths, primary branch localizations, secondary branch numbers/lengths, AChR cluster numbers, and areas of AChR cluster number distribution did not differ between the two groups at either age (Figure 8B–G). In addition, we characterized single NMJs of P0 and adult mice. As expected, the NMJ morphologies did not differ between the control and cKO mice (Figure 8H), and nerve coverage was comparable between the control and cKO mice (Figure 8I). These results indicate that Lrp4 ablation does not influence SC development.

To investigate whether SC-derived Lrp4 is involved in the NMJ transmission, mEPPs, which replicate muscle membrane depolarizations due to spontaneous acetylcholine release, were measured by electrophysiology recordings as described previously [40,41,42]. The resting membrane potentials in the cKO mice were similar to those in the control mice (Figure 8J). As shown in Figure 8K,L, both the frequency and amplitude of mEPPs in the cKO mice were comparable to those in the control mice. Moreover, the EPPs that indicate local electrical responses in response to nerve stimulation were comparable between the control and cKO mice (Figure 8M,N). Taken together, these findings suggest normal NMJ development and maintenance in the SC *Lrp4 cKO* mice.

## 4. Discussion

In this study, we characterized the novel function of Lrp4 in mammalian SC by taking advantage of the specific conditional knockout of Lrp4 in the SCs of *Dhh::cre;Lrp4^f/f^* mice and a reporter gene expressed in Lrp4 LacZ mice. We have shown for the first time that Lrp4 is expressed in SCs. As skeletal muscle fibers are not included in the sciatic nerve, our previous results indicate that motor neurons may contribute equal amounts of Lrp4 protein to SCs [19]. Here, the level of Lrp4 was decreased by approximately 50%, indicating that Lrp4 was successfully deleted from the SCs of Lrp4 cKO mice. Intriguingly, Lrp4 deficiency in SCs was advantageous in response to sciatic nerve injury. SC *Lrp4 cKO* mice exhibited accelerated NMJ regeneration compared with that of the control group. Moreover, the downregulation of Krox-20 may increase the demyelination and proliferation of mSCs upon Lrp4 deficiency in SCs, thereby promoting the regeneration of the PNS.

Initially, Lrp4 was largely known for its intricate role in NMJ formation by participating in the Agrin-Lrp4-MuSK signaling pathway. Ablation of the Lrp4 gene or addition of an anti-Lrp4-antibody will induce serious myasthenia gravis disorder across different organisms and in different model systems [43]. Loss of muscle-specific LRP4 in adult mice has demonstrated that Lrp4 is also important for NMJ maintenance [11]. To date, Lrp4 is known to be involved in a myriad of processes, such as limb anomalies [12,13,14], craniofacial organogenesis [15], kidney malformations [16], adult hippocampal neurogenesis [20], ALS [21], and Aβ clearance in Alzheimer’s disease [22]. Recent evidence has indicated that Lrp4 serves as a necessary retrograde signal for presynaptic differentiation in the NMJ and CNS [9,10]; furthermore, it could modulate glutamatergic transmission and Aβ uptake in astrocytes [22]. In this study, we found a novel role of Lrp4 in the regeneration of PNS in mice, thereby providing a potential therapeutic target for peripheral nerve recovery.

Why Lrp4 promotes axon regeneration in low-vertebrate animals while playing an inhibitory role in mammals remains undetermined? First, this intriguing question may be explained by the evolutionary history of Lrp4. Although the sequence of Lrp4 is well conserved (80% identity), the timing of Lrp4 expression differs between zebrafish and mice during the period of AChR prepatterning. Zebrafish Lrp4 is not expressed in the muscle fiber prior to pioneer axon innervation; thus, AChR prepatterning is more dependent on the Wnt-MuSK signaling pathway [26]. Additionally, unlike the motor axons that fail to stop at the NMJ territory and instead crawl aimlessly across muscle fibers in mice, motor axons display only slight axon branching in the absence of Lrp4 in zebrafish [24]. The Lrp4 in mammals appears to have a stronger inhibitory effect on axon movement than that in zebrafish, which is a result of retrograde signal enhancement during evolution to regulate presynaptic differentiation [24]. Another homologous gene that induces differential axon regeneration was previously reported [44]. Second, the differential axon regeneration abilities of zebrafish and mice may also explain this phenomenon. Zebrafish have a robust regeneration ability in both the CNS [45,46] and PNS [47]. The axons in this laser ablation axon model regenerate faster than those in models of mechanistic injury [48]. The results from zebrafish showed that the pioneer axon across the injured site began to traverse approximately 12 h after injury, and the axons emerging later mostly traversed the injury site at approximately 20 h. Axon regeneration in the PNS generally begins approximately 3–7 days after injury, and broad regeneration occurs in the following several weeks [2]. According to the authors, zebrafish Lrp4 may play function via an extrinsic axon mechanism, through which the released Lrp4 ectodomain binds to the axons to act as a retrograde signal [25]. As axons in zebrafish have a sturdy intrinsic regenerating capacity, this resistance from the Lrp4 adhesion will be turned into the motivation of axons to move forward. However, in mammals, the resistance from the Lrp4 adhesion may be huge, thereby attenuating the speed of axon progression. The differences between zebrafish and mice need to be further researched in the future.

Regarding the mechanism by which Lrp4 promotes axon regeneration, we hypothesize that wild-type Lrp4 may have dual effects on both axon regeneration and SC proliferation, as in the development and maintenance of NMJs in mammals. The expression of wild-type Lrp4 on the SC membrane enhances its adherence to the extracellular matrix, which restricts its mobility and dedifferentiation and thereby reduces its debris clearance and proliferation abilities. In the regrowth cones, as the axon terminal highly expresses adhesion molecules (such as full-length transmembrane Agrin) [49,50], excessive adhesion to the Lrp4 ectodomain prevents regrowth. In Lrp4 ennui mutant zebrafish, in which Lrp4 encodes only the ectodomain, axon regeneration was unaffected [25]. Specifically, the overexpression of Lrp4 in motor neurons does not affect regeneration in zebrafish [25]. However, the downstream molecular mechanism of axon intrinsic factors has not been elucidated, and further research is needed.

A hallmark of SCs is their remarkable plasticity after peripheral nerve injury [51]. In this process, mSCs lose their characteristic myelin-related gene expression pattern. In turn, they start to activate a set of repair-related genes, and the balance between the opposing signaling systems determines the myelination of SCs. After nerve injury, the balance shifts towards the upregulation of myelination negative regulators such as c-Jun and Notch, which trigger SC dedifferentiation and downregulate Krox-20, Mpz, and other proteins positively regulating myelin [36,52,53,54,55,56]. In our study, the expression of the positive myelin regulator Krox-20 was decreased dramatically in *Lrp4 cKO* mice after injury. Concomitantly, the myelin protein Mpz was also decreased, which is consistent with the conclusion that Krox-20 positively regulates Mpz. The decreased expression of myelin-related proteins may underlie the rapid SC demyelination in the mutant mice, thus making it more convenient for SCs to proliferate and clear myelin debris, thereby promoting nerve regeneration. Interestingly, the expression levels of the negative myelin regulators did not differ between the *Lrp4 cKO* and control mice, although they were all upregulated under injury conditions.

## 5. Conclusions

In summary, we first revealed that Lrp4 is expressed in the SCs of mice. Surprisingly, the ablation of Lrp4 in SCs was shown to promote peripheral nerve regeneration. The expression of Krox-20, a transcription factor essential for SC myelination during regeneration, was decreased in SC *Lrp4 cKO* mice, thus driving the demyelination and proliferation of mSCs. Our results reveal a novel role of Lrp4 in peripheral nerve regeneration, and may provide a new therapeutic target for peripheral nerve regeneration.

## Data Availability

All data generated or analyzed during this study are included in this published article.

## References

[1] Zhu S., Ge J., Wang Y., Qi F., Ma T., Wang M., Yang Y., Liu Z., Huang J., Luo Z. (2014). A synthetic oxygen carrier-olfactory ensheathing cell composition system for the promotion of sciatic nerve regeneration. Biomaterial.

[2] Min Q., Parkinson D.B., Dun X. (2021). Migrating Schwann cells direct axon regeneration within the peripheral nerve bridge. Glia.

[3] Morris J.K., Lin W., Hauser C., Marchuk Y., Getman D., Lee K.-F. (1999). Rescue of the Cardiac Defect in ErbB2 Mutant Mice Reveals Essential Roles of ErbB2 in Peripheral Nervous System Development. Neuron.

[4] Jessen K.R., Mirsky R. (2019). The Success and Failure of the Schwann Cell Response to Nerve Injury. Front. Cell. Neurosci..

[5] Darabid H., Arbour D., Robitaille R. (2013). Glial Cells Decipher Synaptic Competition at the Mammalian Neuromuscular Junction. J. Neurosci..

[6] Smith I.W., Mikesh M., Lee Y.I., Thompson W.J. (2013). Terminal Schwann cells participate in the competition underlying neuromuscular synapse elimination. J. Neurosci..

[7] Barik A., Li L., Sathyamurthy A. (2016). Schwann Cells in Neuromuscular Junction Formation and Maintenance. J. Neurosci..

[8] Lee Y.I., Thompson W.J., Harlow M.L. (2017). Schwann cells participate in synapse elimination at the developing neuromuscular junction. Curr. Opin. Neurobiol..

[9] Wu H., Lu Y., Shen C., Patel N., Gan L., Xiong W.C., Mei L. (2012). Distinct Roles of Muscle and Motoneuron LRP4 in Neuromuscular Junction Formation. Neuron.

[10] Yumoto N., Kim N., Burden S.J. (2012). Lrp4 is a retrograde signal for presynaptic differentiation at neuromuscular synapses. Nat. Cell Biol..

[11] Barik A., Lu Y., Sathyamurthy A. (2014). LRP4 is critical for neuromuscular junction maintenance. J. Neurosci..

[12] Leupin O., Piters E., Halleux C., Hu S., Kramer I., Morvan F., Bouwmeester T., Schirle M., Bueno-Lozano M., Fuentes F.J.R. (2011). Bone Overgrowth-associated Mutations in the LRP4 Gene Impair Sclerostin Facilitator Function. J. Biol. Chem..

[13] Bullock W.A., Hoggatt A.M., Horan D.J. (2019). Lrp4 Mediates Bone Homeostasis and Mechanotransduction through Interaction with Sclerostin In Vivo. iScience.

[14] Tian J., Shao J., Liu C., Hou H.-Y., Chou C.-W., Shboul M., Li G.-Q., El-Khateeb M., Samarah O.Q., Kou Y. (2018). Deficiency of lrp4 in zebrafish and human LRP4 mutation induce aberrant activation of Jagged–Notch signaling in fin and limb development. Cell. Mol. Life Sci..

[15] Ohazama A., Porntaveetus T., Ota M.S., Herz J., Sharpe P.T. (2010). Lrp4: A novel modulator of extracellular signaling in craniofacial organogenesis. Am. J. Med. Genet. Part A.

[16] Li Y., Pawlik B., Elcioglu N. (2010). LRP4 mutations alter Wnt/beta-catenin signaling and cause limb and kidney malformations in Cenani-Lenz syndrome. Am. J. Hum. Genet..

[17] Ahn Y., Sims C., Logue J.M., Weatherbee S.D., Krumlauf R. (2013). Lrp4 and Wise interplay controls the formation and patterning of mammary and other skin appendage placodes by modulating Wnt signaling. Development.

[18] Mosca T.J., Luginbuhl D.J., Wang I.E. (2017). Presynaptic LRP4 promotes synapse number and function of excitatory CNS neurons. eLife.

[19] Gomez A.M., Froemke R.C., Burden S.J. (2014). Synaptic plasticity and cognitive function are disrupted in the absence of Lrp4. Elife.

[20] Zhang H., Sathyamurthy A., Liu F., Li L., Zhang L., Dong Z., Cui W., Sun X., Zhao K., Wang H. (2019). Agrin-Lrp4-Ror2 signaling regulates adult hippocampal neurogenesis in mice. eLife.

[21] Tzartos J.S., Zisimopoulou P., Rentzos M., Karandreas N., Zouvelou V., Evangelakou P., Tsonis A., Thomaidis T., Lauria G., Andreetta F. (2013). LRP 4 antibodies in serum and CSF from amyotrophic lateral sclerosis patients. Ann. Clin. Transl. Neurol..

[22] Zhang H., Chen W., Tan Z. (2020). A Role of Low-Density Lipoprotein Receptor-Related Protein 4 (LRP4) in Astrocytic Abeta Clearance. J. Neurosci..

[23] Ye X.-C., Hu J.-X., Li L., Li Q., Tang F.-L., Lin S., Sun N., Sun X.-D., Cui G.-Y., Mei L. (2018). Astrocytic Lrp4 (Low-Density Lipoprotein Receptor–Related Protein 4) Contributes to Ischemia-Induced Brain Injury by Regulating ATP Release and Adenosine-A2AR (Adenosine A2A Receptor) Signaling. Stroke.

[24] DePew A., Mosca T. (2021). Conservation and Innovation: Versatile Roles for LRP4 in Nervous System Development. J. Dev. Biol..

[25] Gribble K.D., Walker L.J., Saint-Amant L., Kuwada J.Y., Granato M. (2018). The synaptic receptor Lrp4 promotes peripheral nerve regeneration. Nat. Commun..

[26] Remédio L., Gribble K.D., Lee J.K., Kim N., Hallock P.T., Delestrée N., Mentis G.Z., Froemke R.C., Granato M., Burden S.J. (2016). Diverging roles for Lrp4 and Wnt signaling in neuromuscular synapse development during evolution. Genes Dev..

[27] Sun X.-D., Li L., Liu F., Huang Z.-H., Bean J.C., Jiao H.-F., Barik A., Kim S.-M., Wu H., Shen C. (2016). Lrp4 in astrocytes modulates glutamatergic transmission. Nat. Neurosci..

[28] Yu Z., Zhang M., Luo B., Jing H., Yu Y., Wang S., Luo S. (2020). Lrp4 in hippocampal astrocytes serves as a negative feedback factor in seizures. Cell Biosci..

[29] Liang C., Tao Y., Shen C., Tan Z., Xiong W.-C., Mei L. (2012). Erbin Is Required for Myelination in Regenerated Axons after Injury. J. Neurosci..

[30] Zhao K., Shen C., Lu Y., Huang Z., Li L., Rand C.D., Pan J., Sun X.-D., Tan Z., Wang H. (2017). Muscle Yap Is a Regulator of Neuromuscular Junction Formation and Regeneration. J. Neurosci..

[31] Kang H., Tian L., Mikesh M., Lichtman J.W., Thompson W.J. (2014). Terminal Schwann Cells Participate in Neuromuscular Synapse Remodeling during Reinnervation following Nerve Injury. J. Neurosci..

[32] Castro R., Taetzsch T., Vaughan S.K., Godbe K., Chappell J., Settlage R.E., Valdez G. (2020). Specific labeling of synaptic schwann cells reveals unique cellular and molecular features. eLife.

[33] Adilakshmi T., Ness-Myers J., Madrid-Aliste C., Fiser A., Tapinos N. (2011). A Nuclear Variant of ErbB3 Receptor Tyrosine Kinase Regulates Ezrin Distribution and Schwann Cell Myelination. J. Neurosci..

[34] Hirata K., Kawabuchi M. (2002). Myelin phagocytosis by macrophages and nonmacrophages during Wallerian degeneration. Microsc. Res. Tech..

[35] Vargas M.E., Watanabe J., Singh S.J., Robinson W.H., Barres B.A. (2010). Endogenous antibodies promote rapid myelin clearance and effective axon regeneration after nerve injury. Proc. Natl. Acad. Sci. USA.

[36] Jessen K.R., Mirsky R. (2008). Negative regulation of myelination: Relevance for development, injury, and demyelinating disease. Glia.

[37] Jessen K.R., Mirsky R. (2002). Signals that determine Schwann cell identity*. J. Anat..

[38] Chen Z.-L., Yu W.-M., Strickland S. (2007). Peripheral Regeneration. Annu. Rev. Neurosci..

[39] Decker L., Desmarquet-Trin-Dinh C., Taillebourg E., Ghislain J., Vallat J.-M., Charnay P. (2006). Peripheral Myelin Maintenance Is a Dynamic Process Requiring Constant Krox20 Expression. J. Neurosci..

[40] Liu Y., Sugiura Y., Padgett D. (2010). Postsynaptic development of the neuromuscular junction in mice lacking the gamma-subunit of muscle nicotinic acetylcholine receptor. J. Mol. Neurosci..

[41] Chen F., Liu Y., Sugiura Y., Allen P.D., Gregg R.G., Lin W. (2011). Neuromuscular synaptic patterning requires the function of skeletal muscle dihydropyridine receptors. Nat. Neurosci..

[42] Zhao K., Shen C., Li L., Wu H., Xing G., Dong Z., Jing H., Chen W., Zhang H., Tan Z. (2018). Sarcoglycan Alpha Mitigates Neuromuscular Junction Decline in Aged Mice by Stabilizing LRP4. J. Neurosci..

[43] Lin M., Xiong W.-C., Mei L. (2018). Neuromuscular Junction Formation, Aging, and Disorders. Annu. Rev. Physiol..

[44] Abdesselem H., Shypitsyna A., Solis G.P. (2009). No Nogo66- and NgR-mediated inhibition of regenerating axons in the zebrafish optic nerve. J. Neurosci..

[45] Zou S., Tian C., Ge S., Hu B. (2013). Neurogenesis of Retinal Ganglion Cells Is Not Essential to Visual Functional Recovery after Optic Nerve Injury in Adult Zebrafish. PLoS ONE.

[46] Diekmann H., Kalbhen P., Fischer D. (2015). Characterization of optic nerve regeneration using transgenic zebrafish. Front. Cell. Neurosci..

[47] Gonzalez D., Allende M. (2021). Current Advances in Comprehending Dynamics of Regenerating Axons and Axon–Glia Interactions after Peripheral Nerve Injury in Zebrafish. Int. J. Mol. Sci..

[48] Hu B.-B., Chen M., Huang R.-C., Huang Y.-B., Xu Y., Yin W., Li L., Hu B. (2018). In vivo imaging of Mauthner axon regeneration, remyelination and synapses re-establishment after laser axotomy in zebrafish larvae. Exp. Neurol..

[49] Magill-Solc C., Mcmahan U.J. (1988). Motor neurons contain agrin-like molecules. J. Cell Biol..

[50] Neuhuber B., Daniels M.P. (2003). Targeting of recombinant agrin to axonal growth cones. Mol. Cell. Neurosci..

[51] Suzuki K., Lovera M., Schmachtenberg O., Couve E. (2015). Axonal Degeneration in Dental Pulp Precedes Human Primary Teeth Exfoliation. J. Dent. Res..

[52] De Felipe C., Hunt S.P. (1994). The differential control of c-jun expression in regenerating sensory neurons and their associated glial cells. J. Neurosci..

[53] Parkinson D.B., Bhaskaran A., Droggiti A., Dickinson S., D’Antonio M., Mirsky R., Jessen K.R. (2004). Krox-20 inhibits Jun-NH2-terminal kinase/c-Jun to control Schwann cell proliferation and death. J. Cell Biol..

[54] Parkinson D.B., Bhaskaran A., Arthur-Farraj P. (2008). c-Jun is a negative regulator of myelination. J. Cell Biol..

[55] Matsuno K. (2019). Notch signaling. Dev. Growth Differ..

[56] Jessen K.R., Mirsky R. (2016). The repair Schwann cell and its function in regenerating nerves. J. Physiol..

